# Clinical value of serum AFP and PIVKA‐II for diagnosis, treatment and prognosis of hepatocellular carcinoma

**DOI:** 10.1002/jcla.25016

**Published:** 2024-02-23

**Authors:** Yujiao Jin, Aifang Xu

**Affiliations:** ^1^ Department of Clinical Laboratory Xixi Hospital of Hangzhou Hangzhou China

Dear Editor,

We read with great interest the paper by Tian et al.^1^ titled “Clinical value of serum AFP and PIVKA‐II for diagnosis, treatment and prognosis of hepatocellular carcinoma.” We congratulate Tian et al. for the excellent research, but some issues of the research warrant discussion.

In figure 2a, b in Ref. [1], serum AFP and PIVKA‐II levels were compared pairwise among the five groups by rank sum test according to the introduction of statistical methods. Multiple testing in the five groups increased the chance of making type I errors; however, Tian et al. did not pay attention to this matter in their paper. There are many different methods to control type I errors in multiple testing, such as Bonferroni correction by controlling the family‐wise error rate and the Benjamini–Hochberg procedure by controlling the false discovery rate.^2^, ^3^

The area under the receiver operating characteristic curve (AUROC) was calculated to assess diagnostic performance. Tian et al. found that the diagnostic value of the combined detection of AFP and PIVKA‐II was greater than that of AFP and PIVKA‐II alone, because the AUROC of the combination of AFP and PIVKA‐II (0.975, 95% CI 0.956–0.994) was greater than that of AFP (0.903, 95% CI 0.862–0.944) and PIVKA‐II (0.945, 95% CI 0.915–0.975) in comparison with HCC and benign liver disease. This conclusion appears to be arbitrary. It seems to be more rigorous that the statistical significance of difference between two AUROCs was evaluated by the Delong test.^4^

Despite the above‐mentioned potential limitations, Tian et al. have made several contributions in assessing the role of serum AFP and PIVKA‐II in the diagnosis, treatment, and prognosis of HCC.

## CONFLICT OF INTEREST STATEMENT

The authors have no conflicts of interest to declare.
